# Diversity and selective pressures of anticoagulants in three medicinal leeches (Hirudinida: Hirudinidae, Macrobdellidae)

**DOI:** 10.1002/ece3.480

**Published:** 2013-03-05

**Authors:** Sebastian Kvist, Gi-Sik Min, Mark E Siddall

**Affiliations:** 1 Richard Gilder Graduate School, American Museum of Natural HistoryCentral Park West at 79th Street, New York, NY 10024, USA; 2 Department of Biological Sciences, Inha UniversityYonghyun-dong, Nam-Gu, Incheon 402-751, Korea; 3 Division of Invertebrate Zoology, American Museum of Natural HistoryCentral Park West at 79th Street, New York, NY 10024, USA; 4 Sackler Institute for Comparative Genomics, American Museum of Natural HistoryCentral Park West at 79th Street, New York, NY 10024, USA

**Keywords:** Anticoagulant, evolution, FEL, Hirudinida, medicinal leeches, PARRIS, REL, selection pressures, SLAC

## Abstract

Although medicinal leeches have long been used as treatment for various ailments because of their potent anticoagulation factors, neither the full diversity of salivary components that inhibit coagulation, nor the evolutionary selection acting on them has been thoroughly investigated. Here, we constructed expressed sequence tag libraries from salivary glands of two species of medicinal hirudinoid leeches, *Hirudo verbana* and *Aliolimnatis fenestrata*, and identified anticoagulant-orthologs through BLASTx searches. The data set then was augmented by the addition of a previously constructed EST library from the macrobdelloid leech *Macrobdella decora*. The identified orthologs then were compared and contrasted with well-characterized anticoagulants from a variety of leeches with different feeding habits, including non-sanguivorous species. Moreover, four different statistical methods for predicting signatures of positive and negative evolutionary pressures were used for 10 rounds each to assess the level and type of selection acting on the molecules as a whole and on specific sites. In total, sequences showing putative BLASTx-orthology with five and three anticoagulant-families were recovered in the *A. fenestrata* and *H. verbana* EST libraries respectively. Selection pressure analyses predicted high levels of purifying selection across the anticoagulant diversity, although a few isolated sites showed signatures of positive selection. This study represents a first attempt at mapping the anticoagulant repertoires in a comparative fashion across several leech families.

## Introduction

Notwithstanding that the documented use of leeches for medicinal purposes dates back over two millennia, emphasis on the utility of leeches in modern medicine is becoming more authoritative (Whitaker et al. 2004; Phillips and Siddall 2009; Min et al. 2010). The most conspicuous application of leeches is that for relief of venous congestion following flap and digit replantation surgery (Dabb et al. 1992; Soucacos et al. 1994). Essential to this application are leech anticoagulants, proteins that interfere with a normal thrombus formation at various stages of the coagulation cascade, and that play an important role in the leeches' ability to feed for extended periods. Most widely exploited of these is hirudin, first extracted from the European medicinal leech *Hirudo medicinalis* Linnaeus, 1758. Hirudin binds irreversibly to the fibrinogen exosite of thrombin as well as to the catalytic pocket (Rydel et al. 1990) and, with an inhibition constant in the picomolar range, it remains the most potent natural direct thrombin inhibitor known (Greinacher & Warkentin 2008). However, leech salivary glands produce a more diverse pharmacological cocktail of a wide variety of anticoagulants (e.g., Min et al. 2010; Alaama et al. 2011) that not only assist in phlebotomy by keeping blood flowing in and around an incision wound but that also keeps the blood from coagulating inside the leech crop during the long periods of digestion (Salzet 2001). As an example of the diversity of coagulation factors targeted by leech anticoagulants, leech antiplatelet protein (LAPP) from *Haementeria officinalis* de Fillippi, 1849, in contrast to hirudin, inhibits von Willebrand factor-mediated, and collagen-stimulated, platelet aggregation by binding to subendothelial collagen (Connolly et al. 1992). Other leech bioactive salivary peptides target (e.g.,) factor Xa, factor XIIIa, plasmin, and hyaluronic acid. Despite the renaissance of leech anticoagulants in medical practices, anticoagulant profiles are known for only three of the more than 800 species.

Whereas the European *Hirudo verbana* Carena, 1820 remains the model for biomedical studies on leeches (not *H. medicinalis* as previously thought; Siddall et al. 2007), much as it is the focal point for several other areas of invertebrate biology (Shain 2009), other continents are inhabited by hirudiniform counterparts equivalent to *Hirudo verbana* in terms of feeding habits. These include *Macrobdella* spp. in North America, *Aliolimnatis* spp. in Africa, *Hirudinaria* spp in Asia, *Goddardobdella* spp. in Australia, and (e.g.,) *Oxyptychus* spp. in South America. Despite the infrequent mention of these leeches in medical contributions, there is some evidence that these leeches historically have been used to treat medical conditions in light of their equivalent bloodfeeding behaviors (Phillips and Siddall 2009). Sanguivory, however, also occurs in several other, only distantly related, leech families including Glossiphoniidae, Piscicolidae, Praobdellidae, Haemadipsidae, and Xerobdellidae (Min et al. 2010). Contemporary studies seem to agree that bloodfeeding is a plesiomorphic strategy in leeches (Siddall and Burreson 1995, 1996; Trontelj et al. 1999; Min et al. 2010) and it has even been demonstrated that at least one non-bloodfeeding leech, *Helobdella robusta* Shankland et al. 1992 (Glossiphoniidae), possesses ancestrally inherited anticoagulants (Kvist et al. 2011). Min et al. (2010) described the partial transcriptome of the North American medicinal leech, *Macrobdella decora* (Say, 1824), and found several loci with very high sequence similarity to eight known anticoagulants in addition to predicted serine protease inhibitors, lectoxin-like c-type lectins, ficolin, disintegrins, and histidine-rich proteins. In the same contribution, the authors conclude that “the goal of identifying evolutionarily significant residues associated with biomedically significant phenomena implies continued insights from a broader sampling of blood-feeding leech salivary transcriptomes.” As such, sampling in a phylogenetic framework and focusing on sanguivorous taxa across the fullness of the leech phylogeny will greatly increase our understanding of the evolution of bloodfeeding in leeches. Moreover, identifying regions under negative and positive evolutionary selection within the anticoagulant molecules holds the potential to highlight functionally critical gene regions, thus providing a more convincing understanding of the structure–function relationships of anticoagulant proteins.

## Material and methods

### Taxon sampling and EST library creation

**Figure 1:**
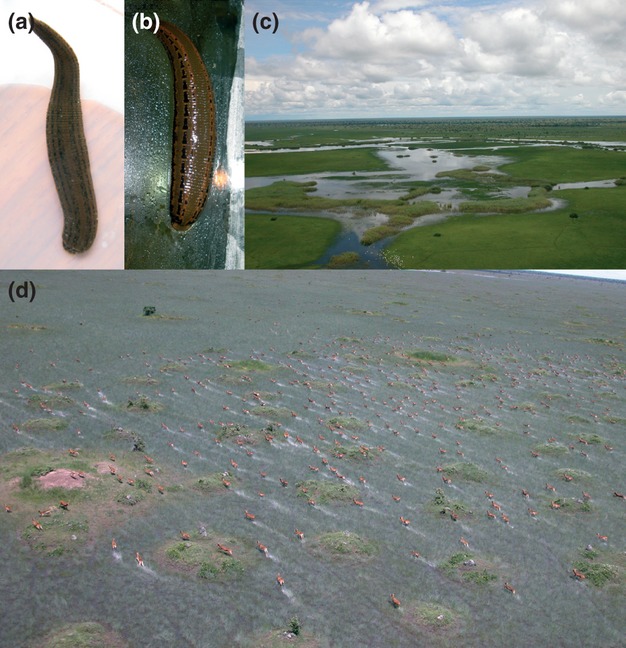
Medicinal leech specimens used for this study and images of the collection sites for *Aliolimnatis fenestrata*. (a) the African medicinal leech *A. fenestrata,* (b) the European medicinal leech *Hirudo verbana*, (c) flooded plains of Kasanka National Park, Zambia, (d) flooded plains of the Bangweulu region, Zambia with a team of black lechwe (*Kobus leche smithemani*), one of the prey of *A. fenestrata*.

Two hirudinoid leeches were chosen for salivary EST library creation: the African medicinal leech *Aliolimnatis fenestrata* (Fig. 1a) and the European medicinal leech *Hirudo verbana* (Fig. 1b). Specimens of *A. fenestrata* were collected in Kasanka National Park, Zambia (Fig. 1 c–d) from exposed skin while wading in ponds, and specimens of *H. verbana* were obtained from Leeches USA Ltd. (Westbury, New York). The data set then was augmented by the addition of a previously constructed EST library for the North American macrobdelloid medicinal leech *M. decora* (Min et al. 2010).

Prior to RNA extraction, leeches were washed in 0.5% bleach for 1 min and rinsed in deionized water for 1 min in order to minimize contamination of surface bacteria. Using sterilized tools, salivary tissue masses (glandular tissue) were removed aseptically by dissection while immersed in RNA*later* (Qiagen, Valencia, California) and subsequently rinsed in 0.5% bleach for 1 min then rinsed in deionized water for 1 min. RNA then was isolated using RNeasy Tissue kit (Qiagen). Subsequent construction of cDNA libraries, as well as low-quality sequence and repeat masking, follows the protocol detailed by Min et al. (2010).

### Similarity and identification using BLASTx

A relational database for all EST sequences from all three species was created in FileMaker Pro (FileMaker, Santa Clara, California) following the removal of low-quality sequences as determined with Sequence Analysis Software ver. 5.4 (Applied Biosystems). At this point, sequences shorter than 150 bp in length also were removed from the data set. Vector and adaptor sequence removal was not necessary due to the use of Smart-seq sequencing primer, which anneals to within 3 bp of the cloned insert. Nonetheless, the first 20–30 bp were automatically trimmed to minimize the inclusion of 5′ sequencing errors. Following Min et al. (2010), sequences then were clustered locally using a BLASTn protocol based on an inclusion criterion of 1E^−5^ expectation value (e-value).

A single, high-quality, non-repetitive sequence from each cluster was employed as a query in a BLASTx search (searching a protein database using a translated nucleotide query) against a locally compiled set of known anticoagulants (Table 1). The BLASTx search used a cutoff e-value of 1E^−5^. Moreover, the anticoagulant data set was queried both against a stand-alone EST database for *H. medicinalis* on the Hirudinea Genomics Consortium website (http://genomes.sdsc.edu/leechmaster/database/) and against the genome of *Helobdella robusta*, available at the Joint Genome Institute (JGI) portal website (http://genome.jgi-psf.org/Helro1/Helro1.home.html). Both searches employed a cutoff e-value of 1E^−5^.

**Table 1:** Anticoagulants included in the locally compiled data set used for the BLASTx comparisons

Species	Anticoagulant	Genbank Accn. number	Reference
*Hirudo medicinalis*	Hirudin	Q07558	Scacheri et al. 1993
*Poecilobdella viridis*	Hirudin	P84590	Vankhede et al. 2005
*Hirudo medicinalis*	Hirudin II	P28504	Scharf et al. 1989
*Hirudinaria manillensis*	Hirulin	P26631	Tulinsky and Qiu 1993
*Hirudo medicinalis*	Bdellin	P09865	Fink et al. 1986
*Hirudo medicinalis*	Destabillase I	AAA96144	Zavalova et al. 1996
*Hirudo medicinalis*	Destabillase II	AAA96143	Zavalova et al. 1996
*Theromyzon tessulatum*	Cystatin	AAN28679	Lefebvre et al. 2004
*Unidentified hirudinid*	Eglin c	0905140A	Knecht et al. 1983
*Haementeria officinalis*	LAPP	Q01747	Keller et al. 1992
*Macrobdella decora*	Decorsin	P17350	Seymour et al. 1990
*Theromyzon tessulatum*	Therostasin	Q9NBW4	Chopin et al. 2000
*Haementeria ghilianii*	Ghilanten	AAB21233	Brankamp et al. 1991
*Haementeria ghilianii*	Ghilanten	P16242	Blankenship et al. 1990
*Haementeria officinalis*	Antistasin	AAA29193	Han et al. 1989
*Haementeria officinalis*	Antistasin	P15358	Han et al. 1989
*Hirudo nipponia*	Guamerin	AAD09442	Jung et al. 1995
*Hirudo medicinalis*	Hirustasin	P80302	Söllner et al. 1994
*Haementeria officinalis*	Saratin	2K13-X	Gronwald et al. 2008
*Hirudinaria manillensis*	Manillase	N/A	US Patent: 2006_US_7.049.124_B1
*Macrobdella decora*	Ficolin	N/A	Min et al. 2010; cluster 686

Sequences matching known anticoagulants were submitted to CodonCode Aligner (Codoncode Corp., Dedham, Massachusetts) where they were reconciled into unigene sequences using a 95% minimum percent identity cutoff and a 25% minimum overlap length between sequences. The longest open-reading frame (ORF) of the single representative (i.e., the “reference” sequence) of the multiple reconciled sequences was recovered. When irreconcilable, the longest ORF for each individual sequence was retained. As a cross-control of the ORF's, reference sequences were translated into amino acids using six-frame translation on the ExPASy Bioinformatics Resource Portal website (http://web.expasy.org/translate/). The longest ORF was confirmed and all sequences were asserted to be in first frame (i.e., the first position of the sequence was the first codon position in all cases). In cases where newly generated EST sequences were substantially longer than the archetypal anticoagulant sequences, these were truncated at the 5′ end, 3′ end, or both. Prediction of signal peptides was performed on the SignalP 4.0 (Petersen et al. 2011) server at http://www.cbs.dtu.dk/services/SignalP/. Jalview ver. 2 (Waterhouse et al. 2009) was used to visualize the alignments in order to assess the level of conservation between translated sequences and protein sequences of the archetypal anticoagulants.

### Phylogenetic analyses

Nucleotide sequences of the unigenes were aligned with their respective known anticoagulant in accordance with their inferred amino acid states by employing Dialign-T (Subramanian et al. 2005) as implemented in RevTrans 1.4 (Wernersson and Pedersen 2003). The alignments for each anticoagulant then were submitted to TNT (Goloboff et al. 2008) for phylogenetic analyses. A traditional search was performed for each data set employing 100 initial addition sequences and TBR branch swapping. All characters were un-weighted and non-additive and gaps were treated as a fifth state. All trees were left unrooted due to the lack of appropriate outgroups.

### Analysis of evolutionary selection

To test the robustness of the inferences made on evolutionary selection, the sequences were also aligned with MUSCLE (Edgar 2004) on the TranslatorX (Abascal et al. 2010) platform website (http://translatorx.co.uk/) in accordance with the amino acid sequences, applying default settings for all parameters and translation used the standard genetic code. Alignments resulting from both Dialign-T and MUSCLE were subjected to selection pressure analyses across the molecule by implementation of the PARRIS method (Scheffler et al. 2006) in HyPhy (Kosakovsky Pond et al. 2005), and for site-specific selection using the codon-based likelihood ratio tests Single-Likelihood Ancestor Counting (SLAC), Fixed Effects Likelihood (FEL), and Random Effects Likelihood (REL). Each anticoagulant alignment for both methods was subjected to 10 rounds of each analysis (PARRIS, SLAC, FEL, and REL), to minimize the effect of type I and type II errors based on stochastic effects. Statistical significance (*P* < 0.05) for ω (or *dN*/*dS*) was assessed in HyPhy and the best fitting models of evolution under the Akaike Information Criterion were predicted using the same software. In addition, HyPhy was used to plot the likelihood ratio test (LRT) scores for each codon position resulting from the FEL analyses.

## Results

### Anticoagulant diversity

After removal of low-quality and repetitive sequences, 1555 and 1800 sequences remained for the EST libraries of *A. fenestrata* and *H. verbana*, respectively; already, 2019 sequences were available for *M. decora* (Min et al. 2010). Sequences from *A. fenestrata* and *H. verbana* were all deposited in the GenBank EST database (accessions JZ183761 - JZ188441). In addition, not all transcripts are accounted for herein, as some of them matched non-anticoagulant proteins exclusively or at better e-values.

For *A. fenestrata,* the 1555 sequences assembled into 408 distinct clusters. The BLASTx search returned hits within the *A. fenestrata* EST library for each of five well-characterized anticoagulant-families, as well as ficolin, eglin c, uncharacterized plasmin inhibitors, leukocyte elastase inhibitors, and lectoxin-like c-type lectin at e-values better than 1E^−5^ (Table 2). Factor Xa-inhibiting proteins (including the leech-isolated antistasin, ghilanten, hirustasin, therostasin, guamerin, and piguamerin) were the most frequently found anticoagulants; 209 of the total 1555 sequences matched antistasin-family proteins at e-values of 1E^−5^ or better. Sequence reconciliation implied one major and one minor unigene transcript. The highest scoring transcript showed an average amino acid identity of 49% when compared with P15358 antistasin from *Haementeria officinalis*, AAB21233 ghilanten from *Haementeria ghilianii*, AAD09442 guamerin from *H. nipponia,* and P80302 hirustasin from *H. medicinalis*. The second most frequently recovered anticoagulant was saratin/LAPP; 102 sequences matched the archetypal sequences at 1E^−5^ or better and these reconciled into three major unigene transcripts. Two additional transcripts, each represented by a single sequence, showed e-values equal to or better than 1E^−5^ when BLASTed against saratin. The transcript with the best e-value displayed 62% amino acid identity when compared with 2K13-X saratin from *Haementeria officinalis*. Putative manillase orthologs comprised six clones in one cluster, all of which reconciled into a single transcript. The percentage of shared amino acid positions between the highest scoring transcript and Patent no. 2006 US 7.049.124 B1 manillase from *Hirudinaria manillensis* was 69%. Furthermore, three irreconcilable putative bdellin orthologs matched the archetypal sequence for bdellin. The shared amino acid identity between the best scoring of these and P09865 bdellin from *H. medicinalis* was 43%. One single sequence representing a putative hirudin ortholog was recovered in the *A. fenestrata* EST library at 9.1E^−31^ (Table 2). This transcript showed 53% amino acid identity, on average, when compared with P28504 hirudin II from *H. medicinalis*, P84590 hirudin from *Poecilobdella viridis,* and P26631 hirulin from *H. manillensis*. Single transcripts significantly matching archetypal sequences were recovered for each of: ficolin (61% amino acid identity with a previously determined ficolin sequence from *M. decora*), eglin c (28% amino acid identity with 0905140A eglin c from an unidentified leech), leukocyte elastase inhibitor (30% amino acid identity with CBBP720 from *H. medicinalis*), and c-type lectin (36% amino acid identity with CN807622 c-type lectin from *Haementeria officinalis*).

**Table 2:** Top anticoagulant BLASTx hits in each of the three EST libraries using a locally compiled set of known anticoagulants as targets

	*Aliolimnatis fenestrata*	*Hirudo verbana*	*Macrobdella decora*
Hirudin	9.1E^−31^	–	1.5E^−7^
Haemadin	2.8E^−12^	–	–
Destabilase I	–	–	1.6E^−60^
Destabilase II	–	–	2.5E^−13^
Saratin	1.7E^−36^	–	2.6E^−45^
Bdellin	4.3E^−11^	1.1E^−6^	2.0E^−6^
Piguamerin	7.3E^−7^	5.6E^−7^	5.6E^−7^
Antistasin	6.0E^−20^	4.8E^−20^	9.5E^−22^
Ghilanten	1.6E^−20^	4.8E^−20^	5.6E^−22^
Hirustasin	3.4E^−19^	8.9E^−6^	–
Therostasin	1.7E^−7^	3.9E^−7^	5.5E^−6^
Ornatin	–	–	4.3E^−7^
LAPP	4.9E^−8^	–	4.8E^−8^
Decorsin	–	–	7.0E^−18^
Ficolin	1.4E^−43^	–	1.0E^−28^
Eglin c	4.5E^−8^	2.2E^−16^	1.9E^−7^
Heparanases	4.5E^−71^	7.0E^−159^	5.7E^−13^
Plasmin inhibitor	1.1E^−12^	1.4E^−12^	4.6E^−9^
Elastase inhibitor	4.0E^−5^	2.1E^−42^	7E^−31^
C-type lectin	2.9E^−13^	7.7E^−8^	6.0E^−12^

For *H. verbana,* the 1800 sequences assembled into 419 clusters. At 1E^−5^, putative orthologs were found in the *H. verbana* EST library for each of three known anticoagulant-families in addition to ficolin, eglin c, plasmin inhibitors, leukocyte elastase inhibitors, and lectoxin-like c-type lectin (Table 2). The most frequently found anticoagulants again belonged to the antistasin-family; these were represented by 14 sequences, which reconciled into three major transcripts. An additional singleton sequence matched antistasin-family proteins at 1E^−5^. The best scoring transcript showed 41% average amino acid identity to the archetypal anticoagulants. A total of 10 sequences, nine of which reconciled into a single unigene, matched bdellin at 1E^−5^ or better. When compared with P09865 bdellin from *H. medicinalis*, the best scoring transcript displayed 51% amino acid identity. Also, single transcripts in the *H. verbana* EST library were found to significantly match the following proteins: manillase (69% similarity with patent no. 2006US7049124B1 manillase from *H. manillensis*), hirudin (average identity of 32% with P28504 hirudin II from *H. medicinalis*, P84590 hirudin from *Poecilobdella viridis,* and P26631 hirulin from *H. manillensis*), eglin c (51% amino acid identity with 0905140A eglin c from an unidentified leech), leukocyte elastase inhibitor (40% identity with CBBP720 from *H. medicinalis*), c-type lectin, and a putative thrombin-inhibiting hirudin-like ortholog (Table 2).

In addition, when screening the stand-alone *H. medicinalis* EST library, sequences showing putative orthology with five anticoagulant-families and five other leech bioactive salivary peptides were found. These included endoglucuronidases, destabilase, bdellin, saratin/LAPP, antistasin, ficolin, leukocyte elastase inhibitors, eglin c, c-type lectin, and cystatin,

### Phylogeny reconstructions and selection pressure analyses

**Figure 2:**
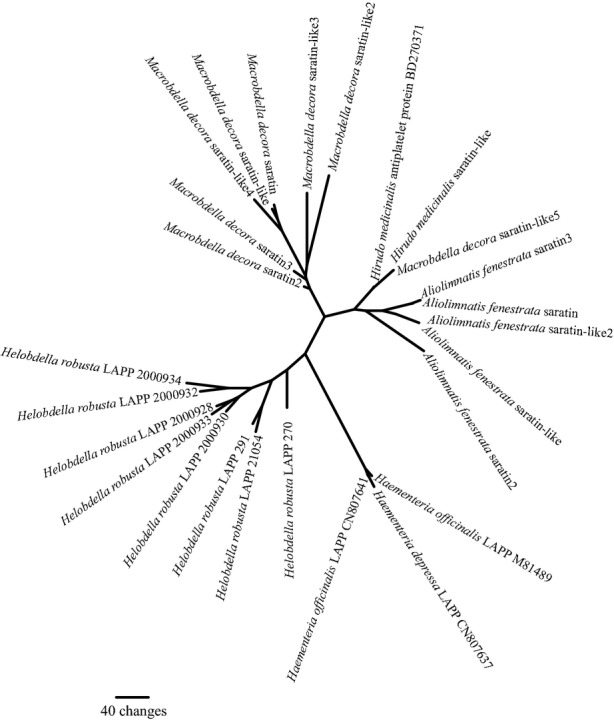
Unrooted strict consensus of two equally parsimonious trees recovered from analysis of the saratin/LAPP data set (see also Table 3)**.** When appropriate, GenBank accession numbers follow taxon names. For *Helobdella robusta* orthologs, the numbers following the taxon name correspond to JGI scaffold for the full genome sequencing. Branch lengths are drawn proportional to change.

**Figure 3:**
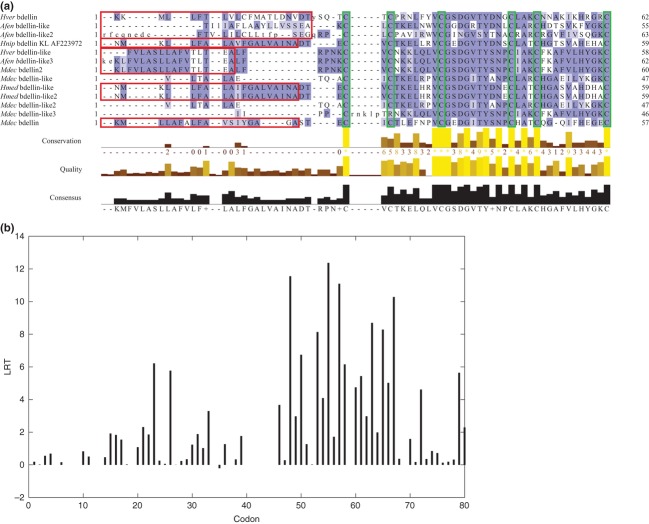
Dialign-T alignment of inferred amino acid sequences for bdellin putative orthologs from *Aliolimnatis fenestrata, Hirudo verbana,* and *Macrobdella decora* together with the archetypal anticoagulant. (a) The full alignment of orthologs across the known taxonomic diversity. Red boxes denote the predicted signal peptide region, green boxes denote fully conserved cysteines, and shading intensity corresponds to BLOSUM62 conservation. Afen, *Aliolimnatis fenestrata*; Hver, *Hirudo verbana*; Mdec, *Macrobdella decora*; Hnip, *Hirudo nipponia*; Hmed, *Hirudo medicinalis*. (b) Likelihood ratio test (LRT) scores for selection pressures at each site, plotted against codon position.

**Figure 4:**
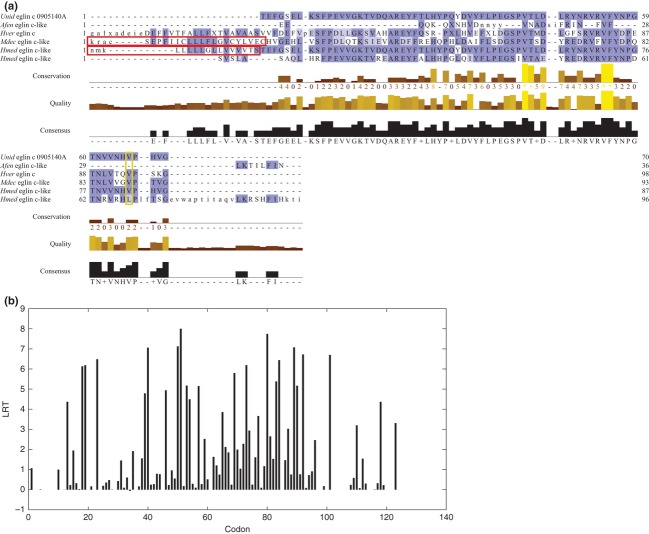
Dialign-T alignment of inferred amino acid sequences for eglin c putative orthologs from *Aliolimnatis fenestrata, Hirudo verbana,* and *Macrobdella decora* together with the archetypal anticoagulants. (a) The full alignment of orthologs across the known taxonomic diversity. Red boxes denote the predicted signal peptide region, yellow boxes denote sites predicted to be under positive selection by REL, and shading intensity corresponds to BLOSUM62 conservation. Unid, unidentified leech; Hver, *Hirudo verbana*; Afen, *Aliolimnatis fenestrata*; Mdec, *Macrobdella decora*; Hmed, *Hirudo medicinalis*. (b) Likelihood ratio test (LRT) scores for selection pressures at each site, plotted against codon position.

Some general statistics for the trees generated from each putatively orthologous data set are presented in Table 3 and the results from the SLAC, FEL, REL, and PARRIS analyses are presented in Table 4. Figure 2 represents the tree derived from the saratin/LAPP putative orthologs and Figs. 3 and 4 represent the Dialign-T amino acid alignments of the bdellin and eglin c data sets, respectively. The remaining trees and amino acid alignments as well as the full results from the selection pressure analyses (including p-values and *dN*/*dS* values) are available as supporting information. The terminology used to describe the unrooted trees shown here follows that of Wilkinson et al. (2007), that is, a “clan” is the equivalent to a “monophyletic group” in a rooted tree and “adjacent group” is equivalent to “sister-group” in a rooted tree.

**Table 3:** Summary statistics of the Dialign-T aligned matrices and resulting phylogenetic trees for each anticoagulant data set. CI, consistency index; RI, retention index

	No. aligned sites	No. parsimony informative characters	No. of most parsimonious trees	Length	CI	RI
Antistasin	627	275	1	2079	0.632	0.642
Saratin/LAPP	669	336	1	2279	0.587	0.740
Hirudin	465	60	1	649	0.924	0.809
Bdellin	240	111	2	565	0.742	0.738
Endoglucuronidases	1257	106	1	2288	0.891	0.852
Decorsin	210	23	1	144	1.000	1.000
Destabilase	882	211	1	453	0.811	0.664
Ficolin	648	132	1	967	0.896	0.646
Eglin c	375	41	1	321	0.938	0.512
Elastase inhibitor	1113	24	1	909	0.987	0.429
C-type lectin	636	153	4	777	0.866	0.388

**Table 4:** Number of codon sites estimated to be under positive or purifying selection in the SLAC, FEL, REL, and PARRIS analyses (*P* ≤ 0.05 in all cases) using the Dialign-T (D) and MUSCLE (M) alignments. In cases where different replicates of the same analysis returned different number of sites under selection, the lowest (most conservative) number is shown. C1 indicates the number of sites agreed upon to be under positive selection by FEL for the two different alignments; C2 indicates the equivalent number for REL; C3, indicates the number of sites under positive selection that are agreed upon by both REL and FEL for both alignments (note that SLAC did not predict any sites under positive selection)

	Significant sites at *P* ≤ 0.05																				
Salivary protein	# Codons		Positive selection								Purifying selection										
						
			SLAC		FEL		REL		PARRIS		SLAC		FEL		REL		PARRIS				
													
	*D*	*M*	*D*	*M*	*D*	*M*	*D*	*M*	*D*	*M*	*D*	*M*	*D*	*M*	*D*	*M*	*D*	*M*			
																			
																			C1	C2	C3
Antistasin	209	182	0	0	1	0	4	0	No	No	21	22	44	39	103	209*	N/A	N/A	0	0	0
Bdellin	80	63	0	0	0	0	0	0	No	No	10	9	17	16	19	19	N/A	N/A	0	0	0
Decorsin	70	70	0	0	0	0	0	0	No	No	0	0	2	12	70*	70*	N/A	N/A	0	0	0
Destabilase	151	144	0	0	1	1	5	14	No	No	15	16	32	31	34	45	N/A	N/A	1	3	1
Eglin c	128	123	0	0	0	0	1	0	No	No	2	5	23	18	35	21	N/A	N/A	0	0	0
Ficolin	216	208	0	0	1	1	0	0	No	No	8	8	44	43	50	46	N/A	N/A	1	0	0
Hirudin	155	135	0	0	0	0	0	0	No	No	1	1	13	11	155*	9	N/A	N/A	0	0	0
Manillase	419	330	0	0	1	0	1	2	No	Yes	3	2	40	25	32	121	N/A	N/A	0	0	0
Saratin	223	200	0	0	2	4	0	7	No	No	11	15	22	21	23	72	N/A	N/A	2	0	0
Elastase inhibitor	371	279	0	0	0	0	0	0	No	No	1	2	32	22	3	1	N/A	N/A	0	0	0
C-type lectin	212	197	0	0	0	2	0	0	No	No	11	11	31	28	26	14	N/A	N/A	0	0	0

* No rates with *dN* > *dS* were inferred, suggesting that all sites are under purifying selection

The analyses agreed on high levels of purifying selection across most of the salivary proteins found in this study and only very few isolated sites showing signs of positive selection. SLAC did not infer positive selection at any site of any salivary protein but, by and large, both the FEL and REL analyses resulted in similar amounts of site-specific positive and negative selection (Table 4). The PARRIS analysis showed evidence of positive selection only for the MUSCLE alignment of the manillase data set. In eight instances did the different rounds of the various analyses result in different codons being predicted to be under positive selection and this always involved the REL method (see supporting information). In the descriptive section below, only positions that were predicted for all 10 rounds are considered.

*Saratin/LAPP*. In the tree (Fig. 2), three main clans can be identified: the first exclusively contains putative orthologs derived from the EST library of *M. decora,* the second includes sequences from only glossiphoniid taxa (*Helobdella robusta* and archetypal sequences from *Haementeria officinalis* and *Haementeria depressa*), and the third includes sequences from each of *A. fenestrata, M. decora,* and *H. medicinalis*. In the latter clan, an antiplatelet protein from *H. medicinalis* (GenBank accession BD270371) is the adjacent group to a saratin-like putative ortholog from *M. decora* as well as a sequence derived from the stand-alone *H. medicinalis* EST library (with very short internal branches).

The FEL analysis of the Dialign-T alignments for the saratin/LAPP data set (Fig. S1a) predicted two codons (100 and 147) to be under positive selection and the same method also inferred positive selection for these sites using the MUSCLE data set. For the latter data set, FEL also predicted two additional codons under positive selection. The principle collagen-binding sites of saratin have been defined (Gronwald et al. 2008) and, interestingly, one of them (Tyr_42_) is predicted by FEL to be under positive selection in the current alignment (this position is also involved in high exchange contributions to conformational motions; Gronwald et al. 2008). The two positively selected codons occur in regions with otherwise high prevalence of negative selection and with high accompanying LRT scores (Fig. S1b). There are particularly high levels of purifying selection in the second domain of the molecule, between codons 98 and 147, including two almost fully conserved lysines (Lys) between the second and third cysteines (Cys).

*Bdellin*. The known sequence of bdellin from *Hirudo nipponia* (GenBank accession AF223972) forms a clan together with two sequences derived from the *H. medicinalis* stand-alone EST library (Fig. S2). In turn, this group places as the adjacent group to a sequence derived from *M. decora*. Beyond this, the remaining sequences are interspersed across the tree without clustering in a taxonomically informative manner.

In both the Dialign-T and MUSCLE alignments for bdellin, putative orthologs show high amino acid conservation compared with the known sequence of the anticoagulant, especially between codons 39 and 80 (corresponding to amino acid positions 23–59 in the archetypal anticoagulant; Fig. 3a). This includes full conservation of six cysteines, assumed to be involved in three disulfide-bonds. No positive selection was predicted for any of the alignments or by any method. The highest LRT scores (Fig. 3b) were retrieved for codon 48 (Lys) and codon 55 (Gly). The identification of two proline (Pro) residues in bdellin from *M. decora* by Min et al. (2010), while surprising (see Fritz et al. 1971), is corroborated by equivalent residues in all variants of putative orthologs in both *H. verbana* and *A. fenestrata*.

*Eglin c*. The sequences derived from *H. medicinalis* form a clan adjacent to a clan containing the remaining sequences from the three EST libraries of *M. decora*, *H. verbana,* and *A. fenestrata* (Fig. S3).

The alignment of the eglin c-like putative orthologs also showed high conservation, in particular between codons 29 and 103 (Fig. 4a) and the FEL analysis of the Dialign-T and MUSCLE alignments predicted 23 and 18 sites as being under purifying selection, respectively. The only codon predicted to be under positive selection was recovered by the REL analysis of the Dialign-T alignment (codon 97). The average LRT score across the alignment is 1.58 (Fig. 4b), the highest (8) occurring at codon 51 (Arg). Our results agree with previous studies (Min et al. 2010) in that no cysteines are present in the eglin c molecules of *A. fenestrata* and *H. verbana*.

*Antistasin*. The known anticoagulants M24423 antistasin from *Haementeria officinalis* and U20787 ghilanten from *Haementeria ghilianii* form a clan positioned as the adjacent group of a larger clan of newly generated sequences from *H. verbana* and *A. fenestrata,* as well as a single sequence from *H. medicinalis* (Fig. S4). By contrast, U38282 guamerin from *H. nipponia* places as the adjacent group to a clade consisting of predicted piguamerin and hirustasin, and putative orthologs from each of *A. fenestrata*, *H. medicinalis, H. depressa,* and *T. tessulatum* (the archetypal sequence for theromyzon).

Although the MUSCLE alignment of the antistasin data set did not predict any positive selection across the alignment, the Dialign-T alignment included five sites under positive selection: codon 26 predicted by FEL and codons 1, 44, 109, and 116 predicted by REL (Fig. S5a). However, the first three of these occur within the predicted signal peptide region, thus outside of any expected active region (Nutt et al. 1988; Dunwiddie et al. 1989). The LRT scores (recovered from the FEL analysis) show a conspicuous spike in the middle of the alignment (Fig. S5b), in a region that is transitively highly conserved. Besides high LRT scores (>10) for the purifying selection acting on disulfide-bond-forming cysteines, there is also high purifying selection acting on a fully conserved glycine (Gly) residue at codon 127, immediately preceding a fully conserved cysteine.

*Hirudin*. The clan of archetypal sequences from *H. medicinalis* (GenBank accessions M12693 and A14988) place as the adjacent group to the *A. fenestrata* EST sequence (Fig. S6). In addition, the thrombin inhibitor and haemadin sequences, both from *Haemadipsa sylvestris* (Z19864 and S58792, respectively), form a clan together with the EST sequence derived from *M. decora*.

No positive selection was inferred for hirudin by any of the three site-specific methods. Transitively, REL for the Dialign-T alignment predicted all sites to be under purifying selection and FEL-LRT scores are high for the prediction of purifying selection across the molecule (Fig. S7a), with two sites in the Dialign-T alignment (codon 52 [Cys] and codon 53 [Glu/Gly]) showing LRT scores above 6 (Fig. S7b). Six cysteines, presumably involved in three disulfide-bonds, are conserved across the alignment, solidifying previous findings of the disulfide-folding pathway of hirudin (Chatrenet and Chang 1993; Min et al. 2010).

*Heparanase-class endoglucuronidases (manillase)*. As nucleotide sequences have yet to be generated for the leech-derived endoglucuronidases manillase and orgelase, an already existing EST sequence (EY484527) showing putative orthology at the amino acid level with manillase acted as the archetypal variant in the endoglucuronidase data set. In the resulting tree (Fig. S8), this sequence is the adjacent group to a clan containing putative orthologs from *H. medicinalis* and *H. verbana*. In turn, this clan places adjacently to the remaining sequences from *A. fenestrata*, *M. decora,* and *H. medicinalis*.

For the site-specific analyses of the Dialignt-T manillase alignment, both FEL and REL predicted a single site to be under positive selection: codon 223 by REL and codon 358 by FEL (Fig. S9a). In addition, the REL analysis of the MUSCLE data set predicted two sites under positive selection, but none of these are agreed upon for both alignment methods. High conservation occurs throughout the alignment; two fully conserved leucines (Leu) at codons 257 and 265 give rise to the two LRT scores above 10 (Fig. S9b).

*Decorsin*. The decorsin data set comprised putative orthologs from *M. decora* and putative orthologs from the stand-alone *H. medicinalis* EST library. The two sequences from the patents for decorsin form a clan and so does the *M. decora* putative orthologs (Fig. S10).

There is no evidence of positive selection acting on the residues in the decorsin data set (Fig. S11a) from either of the alignment methods. The data set included putative orthologs only from *M. decora* as well as the known decorsin sequence. The entire alignment consists of almost fully conserved residues with LRT scores >3 (Fig. S11b) and REL for both alignments predicted all sites as being under purifying selection; the six cysteine residues were fully conserved.

*Destabilase*. Destabilase putative orthologs were only found in *M. decora* and *H. medicinalis*. The archetypal sequence and the *H. medicinalis* ESTs form a clan adajcent to two *M. decora* putative orthologs (Fig. S12). Adjacent to this group is a large clan of sequences derived from *M. decora*.

A single site in the destabilase alignment was predicted to be under positive selection by both FEL and REL under both alignment methods (codon 30 [Ser]). However, this codon is situated within the signal peptide region, thus not in any region of the mature peptide (Fig. S13a). The REL analysis of the Dialign-T alignment further predicts four codons under positive selection and three of these are also predicted by the REL analysis of the MUSCLE alignment. A disproportional spike in LRT score (>15) was calculated for purifying selection acting on the proline (Pro) present at codon 68 (Fig. S13b). Very high sequence conservation is present in the mature peptide (i.e., beyond the signal peptide region) and the number of conserved cysteines (*n* = 14) across the alignment agrees perfectly with previous findings in destabilase (Min et al. 2010).

*Ficolin*. For ficolin, the archetypal sequence, derived from the *M. decora* EST library, places as the adjacent group to two of the three *H. medicinalis* EST sequences; the remaining sequence forms a clan with the single *A. fenestrata* putative ficolin ortholog (Fig. S14).

For ficolin, the FEL analyses for both the Dialign-T and MUSCLE alignments (Fig. S15a), predicted positive selection for the same codon (146). LRT scores (Fig. S15b) are high across the alignment and the two cysteines, putatively involved in a single disulfide-bond, show full conservation.

*Leukocyte elastase inhibitors*. Sequences showing significant matches to leukocyte elastase inhibitors were found in all three species. In the tree (Fig. S16), the *M. decora* putative ortholog places as the adjacent group to the *H. medicinalis* putative ortholog derived from the GenBank EST library, whereas the *H. verbana* and *A. fenestrata* putative orthologs form a clan adjacent to this group.

The alignment of the elastase inhibitor putative orthologs (Fig. S17a) showed high conservation in the last two-thirds, with accompanying high LRT scores (Fig. S17b). No codons where predicted to be under positive selection by any of the methods. The “archetypal” sequence, represented by CBBP720 from *H. medicinalis* (Min et al. 2010), includes five cysteines, whereas sequences from *A. fenestrata* and *H. verbana* do not possess cysteines, and that of *M. decora* only possesses a single cysteine.

*C-type lectin*. Putative c-type lectin orthologs were also found in each of the new EST libraries. In the tree, the two *M. decora* transcripts form a clan with the *H. verbana* transcript, as the adjacent group of a clan that further contains two main clans: the two *H. medicinalis* transcripts and *Haementeria officinalis* + *A. fenestrata* transcripts (Fig. S18).

For the MUSCLE alignment of the c-type lectin data set, FEL predicted two sites under positive selection (codons 25 and 103). This finding was not corroborated by any other method or alignment (Fig. S19a). Amino acid conservation is particularly high in the last section, yet LRT scores vary dramatically across the alignment ranging from 0 to ∼12 (Fig. S19b). Eight fully, or nearly fully, conserved cysteines can be identified, agreeing perfectly with previous findings in c-type lectin (Min et al. 2010).

## Discussion

Our BLAST-annotations of two newly prepared salivary transcriptome EST libraries from the European medicinal leech *H. verbana* and the African medicinal leech *A. fenestrata* show that these leeches each produce pharmacological cocktails with similar salivary protein diversity as that of the North American medicinal leech *M. decora*, with the exception of decorsin and destabilase (expressed salivary proteins in *M. decora* already have been shown to comprise an unprecedented diversity of anticoagulants [Min et al. 2010; ]). The results presented here support the notion that such diversity is general to hirudinoid leeches. In addition, for the three taxa, the frequently predicted signal peptide regions in putatively orthologous sequences, especially for saratin/LAPP, bdellin, antistasin-family proteins, decorsin, destabilase, eglin c, and hirudin are indicative of the secretion of these anticoagulants by the leeches. In contrast to (e.g.,), the functionally diverse and highly prey-specific snake venoms, which commonly evolve under positive selection (Heatwole and Powell 1998; Kordis et al. 1998; Gibbs and Rossiter 2008), maintaining a wide variety of stable anticoagulation factors (mediated by strong purifying selection) would enable even an individual leech to feed on a wide array of prey.

As a first attempt to assess the type and level of selection acting on leech anticoagulants, here we show that purifying selection is extensive across the vast majority of the alignments of the various anticoagulant-like molecules found in this study. In particular, the alignments for saratin/LAPP, antistasin-family proteins and destabilase show very high LRT scores for sites under purifying selection (Figs. S1b, S5b, S13b). Amino acid conservation is rather high throughout the alignments of all the anticoagulants, but polymorphisms and indels do exist and are especially conspicuous in the eglin c, hirudin, and manillase alignments (Fig. 3a, Figs. S7a, S9a). Nonetheless, the importance of the anticoagulant proteins for the leeches appears to be manifested in the high levels of purifying selection, presumably acting on the genes in order to keep the ORF's intact. Common to most of the alignments is the fact that residues adjacent to disulfide-bond-forming cysteines often show high conservation, suggesting their importance for the structure and, by extension, function of the molecule. This is especially evident in the saratin/LAPP, bdellin, decorsin, and destabilase alignments (Figs. S1a, 2a, S11a, S13a), as well as a particular region in the antistasin alignment (Fig. S5a).

Conversely, only a single codon site was predicted to be under positive selection by both FEL and REL simultaneously and across both alignment methods. An additional few sites were predicted to be under positive selection, but none of these were agreed upon by all methods and alignments. Whereas the most conservative test used here (SLAC) did not predict any site to be under positive selection, the FEL analyses for the different alignments agree on sites under positive selection slightly more than the REL analyses do. In fact, FEL agrees on one site in the destabilase alignments, one site in the ficolin alignments, and two sites in the saratin alignments, whereas REL agrees on three sites from the different alignment methods of the destabilase data set. It is important to note that some irreconcilable unigene transcripts were used in this study and, because EST library sequencing is prone to sequencing error (Ulrich et al. 2000; Nagaraj et al. 2007), there is a stronger possibility that these sequences were erroneously inferred. When irreconcilable sequences are excluded from the analyses, one position predicted to be under positive selection by the FEL analyses of the Dialign-T alignment of destabilase changes to neutral selection. This is also the case for all REL-predicted sites under positive selection in the destabilase and eglin c data sets. We opted to include the irreconcilable transcripts as they accounted for a high percentage of the included sequences in these data sets.

Interestingly, for saratin/LAPP and ficolin, positive selection was predicted by FEL for sites situated in regions otherwise characterized by high levels of purifying selection (especially codons 100 and 147 in the saratin/LAPP alignments; Fig. S1a). If these polymorphic sites affect the structure–function relationship of the anticoagulants, this would allow each individual leech to simultaneously target a wide variety of agonists in the coagulation cascades of their prey. In the specific case of saratin/LAPP, it has already been demonstrated that polymorphic orthologs occur as tandem arrays in the genome of the non-bloodfeeding leech *Helobdella robusta*, likely allowing the leeches to simultaneously target the wide assortment of collagen produced by their prey (Kvist et al. 2011). In this study, we identified five different LAPP/saratin unigene transcripts for *A. fenestrata* and eight different unigene transcripts for *M. decora*. No saratin/LAPP putative orthologs were found in the *H. verbana* EST library, but they are known to occur in the closely related *H. medicinalis* (Barnes et al. 2001; Cruz et al. 2001).

Due to both the paucity of comparative data for leech anticoagulant repertoires and the short sequence nature of the peptides, some of the data sets used here may not possess the inherent degrees of freedom needed to accurately infer evolutionary selection pressures (Kosakovsky Pond and Frost 2005). It is also important to note that previous studies have shown that the levels of positive selection can be artificially elevated due to alignment changes or errors (e.g., Wong et al. 2008; Markova-Raina and Petrov 2011). In light of this, it is possible that the sites found to be under positive selection by this study may be inferred as such due to the specifics of the alignment software. To minimize the effects of stochastic errors based on alignment parameters, we used two different alignment softwares and only consider as robustly inferred those sites that are consistently agreed upon by both methods for all rounds of testing. In order to promote a fuller understanding of the selective pressures and diversities of anticoagulation factors (and other bioactive salivary peptides) across the evolutionary history of leeches, future studies should, importantly, appoint both target taxa that widen the scope of diversity and full transcriptome sequencing.

### Decorsin, destabilase, and hirudin: low or transient expression?

Most of the anticoagulants found in this study are common to all three taxa. However, decorsin and destabilase were only found in *M. decora* and hirudin was not recovered in the *H. verbana* EST library. This is notwithstanding that *H. verbana* has been the model for biomedical studies of hirudin for the last 20 years (Salzet 2001; Siddall et al. 2007; Mamelak et al. 2010; Porshinsky et al. 2011; Gröbe et al. 2012). Although the absence of these anticoagulants can be taken as a sign that these are exclusive to certain taxa, it is also possible that they simply were not sequenced here or that they are transiently expressed in the salivary glands. The leech may need excessive or particular stimuli in order to commence the secretion of these proteins or they may only be expressed after a certain time period of bloodfeeding.

### Evolution of anticoagulants

Determining the genealogical relationships of the anticoagulation factors is important for understanding how evolutionary change within each molecule proceeds over time. For most of the anticoagulants, because the putative orthologs from a single species rarely form a clan (*sensu* Wilkinson et al. 2007), the analyses performed here may suggest that these sequences represent several different only homologous (both paralogous and orthologous) loci. This is further corroborated by the irreconcilability of several different transcripts within each taxon, possibly suggesting that these homologs have acquired new functions.

In addition to the phylogeny of the anticoagulants, the leech phylogeny adds a historical correlative framework for inferences on the evolution of the specific leech-derived proteins (Siddall et al. 2011). Overall, however, there is little concordance between the evolutionary histories of the anticoagulants and previous hypotheses of the leech phylogeny (Min et al. 2010; Siddall et al. 2011). In the leech hypotheses, glossiphoniid leeches are recovered at the base of the tree (see also Light and Siddall 1999; Siddall et al. 2005) and *Aliolimnatis* and *Hirudo* are more closely related to each other than either is to *Macrobdella* (see also Phillips and Siddall 2009). The anticoagulant trees shown in this study often show little clan-structure of orthologs from any single species. One exception to this is the tree derived from the saratin/LAPP data set (Fig. 2), which, if rooted at an ortholog from a glossiphoniid taxon, is largely, but not fully, congruent with the reigning leech phylogenetic hypothesis. As mentioned above, the presence of different protein-variants within a single leech (Mason et al. 2004; Faria et al. 2005; Kvist et al. 2011) may be the cause for the lack of concordance between the anticoagulant trees and the leech phylogeny. Regardless of this, our BLAST analyses suggest that, much like Siddall et al. (2011) predicted, the origins of each of hirudin, bdellin, various antistasin-family proteins, eglin c, and endoglucuronidases predate the origins of the medicinal leeches considered here.

## Supporting Information

**Figure S1.** Dialign-T alignment of inferred amino acid sequences for saratin/LAPP putative orthologs from *Aliolimnatis fenestrata* and *Macrobdella decora* together with the archetypal anticoagulants**.** (a) The full alignment of orthologs across the known taxonomic diversity. Red boxes denote the predicted signal peptide regions, green boxes denote fully conserved cysteines, black boxes denote sites predicted to be under positive selection by FEL, and shading intensity corresponds to BLOSUM62 conservation. Afen, *Aliolimnatis fenestrata*; Hver, *Hirudo verbana*; Mdec, *Macrobdella decora*; Hoff, *Haementeria officinalis*; Hrob, *Helobdella robusta*; Hmed, *Hirudo medicinalis*. (b) Likelihood ratio test (LRT) scores for selection pressures at each site, plotted against codon position.

**Figure S2.** Unrooted strict consensus of two equally parsimonious trees recovered from analysis of the bdellin data set (see also Table 3). When appropriate, GenBank accession numbers follow taxon names. Branch lengths are drawn proportional to change.

**Figure S3.** Unrooted single most parsimonious tree recovered from analysis of the eglin c data set (see also Table 3)**.** When appropriate, GenBank accession numbers follow taxon names. Branch lengths are drawn proportional to change.

**Figure S4.** Unrooted single most parsimonious tree recovered from analysis of the antistasin-family data set (see also Table 3). When appropriate, GenBank accession numbers follow taxon names. Branch lengths are drawn proportional to change.

**Figure S5.** Dialign-T alignment of inferred amino acid sequences for antistasin-family putative orthologs from *Aliolimnatis fenestrata, Hirudo verbana,* and *Macrobdella decora* together with the archetypal anticoagulants. (a) The full alignment of orthologs across the known taxonomic diversity. Red boxes denote the predicted signal peptide regions, green boxes denote fully conserved cysteines, black boxes denote sites predicted to be under positive selection by FEL, and yellow boxes denote sites predicted to be under positive selection by REL. Shading intensity corresponds to BLOSUM62 conservation. Afen, *Aliolimnatis fenestrata*; Hver, *H. verbana*; Mdec, *Macrobdella decora*; Hdep, *Haementeria depressa*; Hoff, *Haementeria officinalis*; Hghi, *Haementeria ghilianii*; Hnip, *Hirudo nipponia*; Hmed, *Hirudo medicinalis*; Ttes, *Theromyzon tessulatum*. (b) Likelihood ratio test (LRT) scores for selection pressures at each site, plotted against codon position.

**Figure S6.** Unrooted single most parsimonious tree recovered from analysis of the hirudin data set (see also Table 3)**.** When appropriate, GenBank accession numbers follow taxon names. Branch lengths are drawn proportional to change.

**Figure S7.** Dialign-T alignment of inferred amino acid sequences for hirudin putative orthologs from *Aliolimnatis fenestrata* and *Macrobdella decora* together with the archetypal anticoagulants. (a) The full alignment of orthologs across the known taxonomic diversity. Red boxes denote the predicted signal peptide region and green boxes denote fully conserved cysteines. Shading intensity corresponds to BLOSUM62 conservation. Afen, *Aliolimnatis fenestrata*; Mdec, *Macrobdella decora*; Hmed, *Hirudo medicinalis*; Hsyl, *Hirudinaria sylvestris*. (b) Likelihood ratio test (LRT) scores for selection pressures at each site, plotted against codon position.

**Figure S8.** Unrooted single most parsimonious tree recovered from analysis of the manillase (heparanase-class endoglucuronidase) data set (see also Table 3). When appropriate, GenBank accession numbers follow taxon names. Branch lengths are drawn proportional to change.

**Figure S9.** Dialign-T alignment of inferred amino acid sequences for heparanase-class endoglucuronidase (manillase) putative orthologs from *Aliolimnatis fenestrata, Hirudo verbana,* and *Macrobdella decora* together with the archetypal anticoagulant. (a) The full alignment of orthologs across the known taxonomic diversity. Black boxes denote sites predicted to be under positive selection by FEL and yellow boxes denote sites predicted to be under positive selection by REL. Shading intensity corresponds to BLOSUM62 conservation. Afen, *Aliolimnatis fenestrata*; Hver, *Hirudo verbana*; Mdec, *Macrobdella decora*; Hmed, *Hirudo medicinalis*. (b) Likelihood ratio test (LRT) scores for selection pressures at each site, plotted against codon position.

**Figure S10.** Unrooted single most parsimonious tree recovered from analysis of the decorsin data set (see also Table 3). When appropriate, GenBank accession numbers follow taxon names. Branch lengths are drawn proportional to change.

**Figure S11.** Dialign-T alignment of inferred amino acid sequences for decorsin putative orthologs from *Macrobdella decora* together with the archetypal anticoagulants. (a) The full alignment of orthologs across the known taxonomic diversity. Red boxes denote the predicted signal peptide region and green boxes denote fully conserved cysteines. Shading intensity corresponds to BLOSUM62 conservation. Mdec, *Macrobdella decora*. (b) Likelihood ratio test (LRT) scores for selection pressures at each site, plotted against codon position.

**Figure S12.** Unrooted single most parsimonious tree recovered from analysis of the destabilase data set (see also Table 3). When appropriate, GenBank accession numbers follow taxon names. Branch lengths are drawn proportional to change.

**Figure S13.** Dialign-T alignment of inferred amino acid sequences for destabilase putative orthologs from *Macrobdella decora* together with the archetypal anticoagulant. (a) The full alignment of orthologs across the known taxonomic diversity. Red boxes denote the predicted signal peptide region, green boxes denote fully conserved cysteines, black boxes denote sites predicted to be under positive selection by FEL, and yellow boxes denote sites predicted to be under positive selection by REL. Shading intensity corresponds to BLOSUM62 conservation. Mdec, *Macrobdella decora*; Hmed, *Hirudo medicinalis*. (b) Likelihood ratio test (LRT) scores for selection pressures at each site, plotted against codon position.

**Figure S14.** Unrooted single most parsimonious tree recovered from analysis of the ficolin data set (see also Table 3). When appropriate, GenBank accession numbers follow taxon names. Branch lengths are drawn proportional to change.

**Figure S15.** Dialign-T alignment of inferred amino acid sequences for ficolin putative orthologs from *Aliolimnatis fenestrata* and *Macrobdella decora* together with the archetypal anticoagulant. (a) The full alignment of orthologs across the known taxonomic diversity. Green boxes denote fully conserved cysteines, black boxes denote sites predicted to be under positive selection by FEL, and shading intensity corresponds to BLOSUM62 conservation. Mdec, *Macrobdella decora*; Afen, *Aliolimnatis fenestrata*; Hmed, *Hirudo medicinalis*. (b) Likelihood ratio test (LRT) scores for selection pressures at each site, plotted against codon position.

**Figure S16.** Unrooted single most parsimonious tree recovered from analysis of the leukocyte elastase inhibitor data set (see also Table 3). When appropriate, GenBank accession numbers follow taxon names. Branch lengths are drawn proportional to change.

**Figure S17.** Dialign-T alignment of inferred amino acid sequences for leukocyte elastase inhibitor putative orthologs from *Aliolimnatis fenestrata, Hirudo verbana,* and *Macrobdella decora* together with the archetypal anticoagulant. (a) The full alignment of orthologs across the known taxonomic diversity. Red boxes denote the predicted signal peptide region and shading intensity corresponds to BLOSUM62 conservation. Hver, *Hirudo verbana*; Afen, *Aliolimnatis fenestrata*; Mdec, *Macrobdella decora*; Hmed, *Hirudo medicinalis*. (b) Likelihood ratio test (LRT) scores for selection pressures at each site, plotted against codon position.

**Figure S18.** Unrooted strict consensus of four equally parsimonious tree recovered from analysis of the c-type lectoxin data set (see also Table 3). When appropriate, GenBank accession numbers follow taxon names. Branch lengths are drawn proportional to change.

**Figure S19.** Dialign-T alignment of inferred amino acid sequences for c-type lectin putative orthologs from *Aliolimnatis fenestrata, Hirudo verbana,* and *Macrobdella decora* together with the archetypal anticoagulants. (a) The full alignment of orthologs across the known taxonomic diversity. Red boxes denote the predicted signal peptide region, green boxes denote fully or nearly fully conserved cysteines, and shading intensity corresponds to BLOSUM62 conservation. Hver, *Hirudo verbana*; Afen, *Aliolimnatis fenestrata*; Mdec, *Macrobdella decora*; Hmed, *Hirudo medicinalis*; Hoff, *Haementeria officinalis*. (b) Likelihood ratio test (LRT) scores for selection pressures at each site, plotted against codon position.
